# Different surgical outcome and follow-up status between dMMR and pMMR colorectal cancer patients who fulfilled with Amsterdam-II criteria

**DOI:** 10.1186/s12957-020-01976-8

**Published:** 2020-08-07

**Authors:** Ci-Yuan Sun, Jy-Ming Chiang, Tse-Ching Chen, Hsin-Yun Hung, Jeng-Fu You

**Affiliations:** 1 Division of Colon and Rectal Surgery, Department of Surgery, Chang Gung Memorial Hospital, Lin-Kou Medical Center, Tao-Yuan, Taiwan; 2 College of Medicine, Chang Gung University, No. 5, Fu-Hsing Rd. Kuei-Shan, Tao-Yuan, 333 Taiwan; 3 Chang Gung University, College of Medicine, Tao-Yuan, Taiwan; 4 Department of Pathology, Chang Gung Memorial Hospital, Lin-Kou Medical Center, Tao-Yuan, Taiwan

**Keywords:** Mismatch repair gene expression, Hereditary non-polyposis colorectal cancer, Metachronous colon tumor, Extra-colonic tumors

## Abstract

**Background:**

Although hereditary non-polyposis colorectal cancer (HNPCC) could be subtyped into proficient or deficient mismatch repair gene expression (pMMR or dMMR), distinct clinical features between these two subgroups patients were rarely reported.

**Methods:**

We retrospectively analyzed 175 hereditary non-polyposis colorectal cancer (HNPCC) patients between January 1995 and December 2012. Cox proportional hazards model was used to compare the differences between two subgroups.

**Results:**

Significant differences of disease free survival (DFS) and overall survival (OS) exist between dMMR and pMMR. In addition to other factors including younger mean age of diagnosis for dMMR patients (48.6 years vs. 54.3 years), operation type (more extended colectomy for dMMR 35.8% vs. 14.5%), tumor location (right colon predominance for dMMR 61.7% vs. 27.3% and more rectum cases for pMMR 41.8% vs. 11.7%), tumor differentiation (more poor differentiation for dMMR 23.3% vs. 9.0%), N staging (more N0 cases for dMMR 70.8% vs. 50.9%), more frequently presence of extra-colonic tumors for dMMR (16.7% vs.1.8%), and lower recurrence rates (9.1% vs.35.3%). Significantly different cumulative incidences of developing metachronous colorectal cancer were observed with 6.18 for pMMR patients and 20.57 person-years for dMMR patients (*p* < 0.001).

**Conclusions:**

Distinct clinicopathological features significantly exist between dMMR and pMMR subtypes patient, MMR status should be consider to tailor operation types and follow up surveillance between these two subgroups patients who all fulfilled with Amsterdam-II criteria.

## Background

Patients with hereditary non-polyposis colorectal cancer (HNPCC) may be stratified into Lynch syndrome (LS) and familial colorectal cancer type X (FCCTX) based on the immunohistochemical staining of mismatch repair (MMR) protein expression or microsatellite instability analysis [1]. Patients with deficient mismatch repair gene expression (dMMR) or LS are reported to have certain clinicopathological characteristics including a higher risk of metachronous colorectal tumors [2], younger age at onset, right-side colon predominance, higher proportion of poorly differentiated tumors, mucinous adenocarcinoma [2–5], and a specific tumor lymphocyte infiltration pattern [3]. These histopathologic characteristics may not be shared by patients with proficient mismatch repair gene expression (pMMR) or familial colorectal cancer type X [1, 6]. In addition, patients with FCCTX do not have an increased risk of extra-colonic cancers [6]. Different clinical features were reported. However, differences in surgical outcomes and operation types between these two subgroups of patients have rarely been reported.

For patients with dMMR or Lynch syndrome, extended colectomy such as subtotal or total colectomy, rather than hemicolectomy or segmental resection, is recommended due to the increased risk of metachronous colorectal cancer (CRC) [7, 8]. Performing the recommended surgical intervention has been shown to significantly decrease the rate of metachronous colorectal lesions [2, 9–11]; however, the survival rate did not significantly improve for the extended colectomy subgroups. These patients might die from secondary tumors; we questioned whether extended colectomy was necessary for all HNPCC patients with dMMR as well. In this retrospective study, we analyzed 175 patients with HNPCC, including 120 with dMMR and 55 with pMMR, to investigate whether there are differences in surgical outcomes and post-operation follow-up status between dMMR and pMMR subgroups.

## Methods

### Patients and methods

Between January 1995 and December 2012, 14479 patients underwent colectomy for CRC and were recorded in the CRC Registry of Chang Gung Memorial Hospital. Of these, 175 patients who fulfilled the Amsterdam-II criteria (A-II C) were retrieved. The CRC revised computerized registry was established in 1995 in Chang Gung Memorial Hospital. This database includes records of detailed family histories, demographic variables, preoperative evaluation, surgery, and postoperative follow-up [12]. Patients fulfilling the A-II C (at least three relatives with a Lynch-associated cancer, one being a first-degree relative of the other two; at least two successive generations affected; and at least one person diagnosed before 50 years of age) were defined as having HNPCC.

We adopted neoadjuvant radiotherapy or chemoradiation for high-risk rectal cancer and low rectal cancer. This decreased the local recurrence rate but had no significant effect on distant metastases. Postoperative adjuvant chemotherapy was adopted for all stage III colon cancers and high-risk stage II colon cancers. Postoperative follow-up surveillance included colonoscopy 1 year after colectomy, and then every 1 to 3 years determined on an individual basis according to the status of previous colonoscopic findings. Annual CT for the first three postoperative years was recommended by the NCCN and ESMO guidelines. Some surgeons strictly followed this approach as part of regular follow-up. However, in our hospital, a modified CEA follow-up protocol was adopted in our study. CEA level was measured every 3~4 months in the first 2 years after the operation and every 6 months thereafter in the following 3 years. We performed abdominal CT for patients with elevated CEA level during follow-up since elevation in CEA level may precede clinical symptoms of recurrence for colorectal cancer patients. We also performed CT for patients with abnormal physical examination, such as palpable abdominal mass. Follow-up data were added annually by reviewing patients’ records in medical charts. A telephone interview or mail questionnaire was conducted if a patient’s medical records were not available. The study was approved by the IRB of Chang Gung Memorial Hospital (IRB102-2284B).

### IHC analysis for MMR protein expression

Paraffin-embedded tumor blocks from HNPCC patients were retrieved from the Pathology Department of Chang Gung Memorial Hospital. For each patient, 4-μm-thick sections from one formalin-fixed, paraffin-embedded tissue block containing both tumor tissue and normal adjacent mucosa were obtained. Immunostaining was performed using a Dako Universal Autostainer (DakoCytomation, Denmark) by using ChemMateTM EnvisonTM + Detection kits (DakoCytomation, Denmark) as previously described [12].

### Assessment of MMR protein expression

For the evaluation of IHC results, abnormal staining was defined as total loss of protein in the tumor, using appropriate controls; staining was considered assessable when the nucleus was stained in cells serving as internal controls, including either stromal or germinal follicle lymphocytes or normal epithelial cells in the crypt bases. Tumors were considered negative for MMR protein expression when neoplastic cells showed complete absence of detectable nuclear staining in a sample for which internal positive controls were stained. A pathologist who had no knowledge of the family history or other clinicopathological features, reviewed all cases to confirm the immunostaining results.

### Statistical analyses

Pearson’s chi-square, Fisher’s exact, and the Wilcoxon rank-sum tests were used to evaluate the distribution of patient characteristics between patients with LS and FCCTX. Survival curves were estimated using the Kaplan–Meier method. In univariate survival analysis, the associations between patient characteristics and disease-free survival (DFS) and overall survival (OS) were evaluated using the log-rank test. The Cox proportional hazards model was used to investigate the effect of patient characteristics on survival while adjusting for other explanatory variables. The rate of metachronous CRC was calculated as the number of secondary cancers divided by the number of observed person-years during the follow-up period. In order to explore the association between patient subgroups and risk of secondary cancer occurrence, the risk ratio of the cumulative incidence of secondary malignancies was also estimated using the Cox proportional hazards model. All statistical analyses were performed using SPSS 17 software (SPSS, Inc., Chicago, IL). The *p* values were two-sided and those < 0.05 were considered statistically significant.

## Results

Overall, 68.6% (120/175) of the HNPCC patients demonstrated loss of at least one MMR protein. The distribution of the loss of MMR protein expression included concordant losses of MLH1/PMS2 staining (83 patients, 69.2%), MSH2/MSH6 staining (33 patients, 27.5%), and loss of PMS2 only (4 patients, 3.3%). As shown in Table 1, there were several significant differences between dMMR and pMMR patients in terms of clinical and histopathological characteristics: mean age of diagnosis (younger for dMMR patients 48.6 vs. 54.3 years), operation type (more extended colectomy for dMMR 35.8% vs. 14.5%), tumor location (right colon predominance for dMMR 61.7% vs*.* 27.3%, and more rectum cases for pMMR 41.8% vs. 11.7%), tumor differentiation (more poor differentiation for dMMR 23.3% vs. 9.0%), N stage (more N0 cases for dMMR 70.8% vs. 50.9%), presence of extra-colonic tumors (more frequent for dMMR 16.7% vs*.*1.8%), and recurrence rate (lower for dMMR 9.1% vs. 35.3%). Although the crude rate of metachronous CRC was not significantly different (8.3% vs. 1.8%, *p* = 0.177), the cumulative incidence of developing metachronous CRC was different (6.18 for pMMR patients and 20.57 person-years for dMMR patients; *p* < 0.001).

**Table 1:** Clinicopathological characteristics of HNPCC patients, and comparisons of between dMMR and pMMR patients

Characteristics	All patients (*N* = 175)	HNPCC		*p* value
		pMMR (*n* = 55)	dMMR (*n* = 120)	
Age				
Mean (SD)	50.41 (12.98)	54.31 (13.66)	48.62 (12.30)	0.007
Median (range)	49 (26~88)	51 (29~88)	48 (26~78)	0.017
Sex				0.054
F	92 (52.57)	23 (41.82)	69 (57.50)	
M	83 (47.43)	32 (58.18)	51 (42.50)	
OP type				0.004
Segmental colectomy	124 (70.86)	47 (85.45)	77 (64.17)	
Extended colectomy	51 (29.14)	8 (14.55)	43 (35.83)	
Tumor location				< 0.001
Right colon	89 (50.86)	15 (27.27)	74 (61.67)	
Left colon	49 (28.00)	17 (30.91)	32 (26.67)	
Rectum	37 (21.14)	23 (41.82)	14 (11.67)	
Tumor histology				0.402
Adenocarcinoma	146 (83.43)	49 (89.09)	97 (80.83)	
Mucinous/signet ring	25 (14.29)	5 (9.09)	20 (16.67)	
Unclassified	4 (2.29)	1 (1.82)	3 (2.50)	
Tumor differentiation				0.065
Well	30 (17.14)	12 (21.82)	18 (15.00)	
Moderate	109 (62.29)	38 (69.09)	71 (59.17)	
Poor	33 (18.86)	5 (9.09)	28 (23.33)	
Unclassified	3 (1.71)	0 (0.00)	3 (2.50)	
TNM_T stage				0.421
1	13 (7.51)	5 (9.43)	8 (6.67)	
2	14 (8.09)	3 (5.66)	11 (9.17)	
3	85 (49.13)	30 (56.60)	55 (45.83)	
4	61 (35.26)	15 (28.30)	46 (38.33)	
TNM_N stage				0.049
0	112 (64.74)	27 (50.94)	85 (70.83)	
1	37 (21.39)	15 (28.30)	22 (18.33)	
2	20 (11.56)	10 (18.87)	10 (8.33)	
3	4 (2.31)	1 (1.89)	3 (2.50)	
TNM_M stage				> 0.99
0	161 (92.00)	51 (92.73)	110 (91.67)	
1	14 (8.00)	4 (7.27)	10 (8.33)	
Disease recurrence				< 0.001
No	133 (76.00)	33 (64.70)	100 (90.90)	
Yes	42 (24.00)	18 (35.30)	10 (9.10)	
Extra-colonic tumor				0.005
Absent	154 (88.00)	54 (98.18)	100 (83.33)	
Present	21 (12.00)	1 (1.82)	20 (16.67)	
Metachronous colon tumor				0.177
Absent	146 (83.43)	54 (98.18)	110 (91.67)	
Present	29 (16.57)	1 (1.82)	10 (8.33)	
Death				0.003
Alive	128 (73.14)	32 (58.18)	96 (80.00)	
Deceased	47 (26.86)	23 (41.82)	24 (20.00)	
Follow-up month	97.08 (0.00~239.93)	69.78 (0.00~137.95)	117.47 (0.00~239.93)	0.014

The operation type (extended colectomy vs. segmental colectomy) was significantly associated with the subtype of HNPCC and tumor location. Patients who underwent segmental colectomy were more likely to have tumors in the rectum compared to those who underwent extended colectomy (34/37, 91.9% vs. 3/37, 8.1%; *p* = 0.005) (Table 2) compared to tumors located in the right or left colon. In addition, the rate of extended colectomy was significantly different between dMMR and pMMR patients (35.8% vs. 14.5%, *p* = 0.004) (Table 2).

**Table 2:** Factors related to operation types

Characteristics	All patients *n* (%)	Operation type		*p* value
		Hemicolectomy	Sub/total colectomy	
HNPCC				0.004
pMMR	55 (31.43)	47 (85.45)	8 (14.55)	
dMMR	120 (68.57)	77 (64.17)	43 (35.83)	
Age				**-**
Mean (SD)	50.41 (12.98)	51.77 (13.77)	47.10 (10.21)	
Median (range)	49 (26 - 88)	49 (26 - 88)	48 (29 - 75)	
Sex				0.466
F	92 (52.57)	63 (68.48)	29 (31.52)	
M	83 (47.43)	61 (73.49)	22 (26.51)	
Tumor location				0.005
Right colon	89 (50.86)	60 (67.42)	29 (32.58)	
Left colon	49 (28.00)	30 (61.22)	19 (38.78)	
Rectum	37 (21.14)	34 (91.89)	3 (8.11)	
Tumor histology				0.212
Adenocarcinoma	146 (83.43)	107 (73.29)	39 (26.71)	
Mucinous/signet ring	25 (14.29)	15 (60.00)	10 (40.00)	
Unclassified	4 (2.29)	2 (50.00)	2 (50.00)	
Tumor differentiation				0.265
Well	30 (17.14)	22 (74.31)	8 (26.67)	
Moderate	109 (62.29)	81 (74.31)	28 (25.69)	
Poor	33 (18.86)	19 (57.58)	14 (42.42)	
Unclassified	3 (1.71)	2 (66.67)	1 (33.33)	
TNM_T stage				0.367
1	13 (7.51)	9 (69.23)	4 (30.77)	
2	14 (8.09)	9 (64.29)	5 (35.71)	
3	85 (49.13)	65 (76.47)	20 (23.53)	
4	61 (35.26)	39 (63.93)	22 (36.07)	
TNM_N stage				0.403
0	112 (64.74)	75 (66.96)	37 (35.04)	
1	37 (21.39)	27 (72.97)	10 (27.03)	
2	20 (11.56)	17 (85.00)	3 (15.00)	
3	4 (2.31)	3 (75.00)	1 (25.00)	
TNM_M stage				0.069
0	161 (92.00)	111 (68.94)	50 (31.06)	
1	14 (8.00)	13 (92.86)	1 (7.14)	
TNM staging				0.143
I/II	111 (63.43)	75 (67.57)	36 (32.43)	
III	50 (28.57)	36 (72.00)	14 (28.00)	
IV	14 (8.00)	13 (92.86)	1 (7.14)	

In this study, the average follow-up duration for all patients was 97.1 months and was 69.8 and 117.5 months for pMMR and dMMR patients, respectively. DFS and OS were compared between dMMR and pMMR patients in terms of different clinicopathological variables. Univariate analysis revealed significant differences in DFS based on subtype of HNPCC (dMMR vs. pMMR), type of operation (extended or segmental colectomy), tumor location (right colon, left colon, or rectum), and presence of lymph node metastases (N staging) (Table 3). Multivariate analysis confirmed the significant differences in DFS for subtype of HNPCC (dMMR better than pMMR) and N staging (N2/3 worse than N0) (Table 3). Univariate analysis revealed significant differences in OS based on HNPCC subtype (dMMR and pMMR), type of colectomy, tumor location, and N staging (Table 4); however, in multivariate analysis, a significantly better OS was only found between HNPCC types (dMMR better than pMMR) and N staging (N2/n3 worse than N0) after adjusting for confounding factors.

**Table 3:** Univariate and multivariate analyses of disease-free survival (DFS) related to clinical variables

Characteristics	*N*	DFS (%)			*p* value	Multiple Cox		
		1 year	3 years	5 years		HR	95% C.I.	*p*
HNHPCC					< 0.001			
pMMR	51	84.00	67.94	63.40		1		
dMMR	110	96.25	94.37	93.42		0.28	(0.108~0.733)	0.009
Sex					0.147			
F	86	95.18	89.14	86.65				
M	75	89.13	82.27	0.00				
Operation type					0.029			
Hemicolectomy	111	88.75	80.24	78.27		1		
Sub/total colectomy	50	100.00	98.00	96.00		0.41	(0.113~1.523)	0.185
Tumor location					< 0.001			
Right colon	82	97.48	93.67	93.67		1		
Left colon	48	89.58	85.42	81.14		1.93	(0.601~6.212)	0.269
Rectum	31	82.76	65.52	62.07		1.97	(0.581~6.663)	0.276
Tumor histology					0.522			
Adenocarcinoma	134	92.34	85.41	83.03				
Mucinous/signet ring	23	95.45	90.91	90.91				
Unclassified	4	75.00	75.00	75.00				
Tumor differentiation					0.360			
Well	29	100.00	96.55	92.69				
Moderate	100	89.75	82.52	80.41				
Poor	29	92.86	85.71	85.71				
TNM_T stage					0.678			
1	13	100.00	92.31	92.31		1		
2	14	92.86	92.86	92.86		0.58	(0.052~6.567)	0.662
3	82	91.22	83.61	79.67		0.75	(0.147~3.786)	0.724
4	52	92.00	86.00	86.00		0.86	(0.154~4.773)	0.861
TNM_N stage					< 0.001			
0	111	98.13	92.52	91.54		1		
1	33	93.84	90.61	87.25		1.10	(0.314~3.873)	0.879
2	16	50.00	37.50	31.25		11.22	(4.015~31.343)	< 0.001
3	1	100.00	**–**	**–**		125.71	(9.975~1584.441)	< 0.001

**Table 4:** Univariate and multivariate analyses of overall survival (OS) related to clinical variables

Characteristics	*N*	OS (%)			*p* value	Multiple Cox		
		1 years	3 years	5 years		HR	95% C.I.	*p*
NHPCC					< 0.001			
pMMR	55	92.59	74.07	64.51		1		
dMMR	120	94.95	88.97	87.25		0.48	(0.239~0.966)	0.040
Sex					0.548			
F	92	94.57	85.78	81.38				
M	83	93.81	82.55	78.7				
Operation type					0.006			
Hemicolectomy	124	91.8	78.45	73.4		1		
Sub/total colectomy	51	100	98.04	96.04		0.57	(0.242~1.348)	0.201
Tumor location					< 0.001			
Right colon	89	94.25	88.36	86.01		1		
Left colon	49	97.96	91.84	89.8		1.25	(0.568~2.731)	0.584
Rectum	37	89.19	64.86	53.51		1.42	(0.629~3.217)	0.397
Tumor histology					0.281			
Adenocarcinoma	146	95.17	83.98	79.04				
Mucinous/signet ring	25	91.67	87.5	87.5				
Unclassified	4	75	75	75				
Tumor differentiation					0.276			
Well	30	100	100	92.98				
Moderate	109	93.57	81.53	76.89				
Poor	33	90.63	80.92	80.92				
Unclassified	3	100	66.67	66.67				
TNM_T stage					0.255			
1	13	100	100	83.92				
2	14	92.86	92.86	92.86				
3	85	98.81	84.17	81.69				
4	61	88.52	80.33	75.41				
TNM_N stage					< 0.001			
0	112	97.3	92.79	89.16		1		
1	37	97.3	88.71	82.78		1.18	(0.504~2.757)	0.704
2	20	85	50	45		4.70	(2.063~10.713)	< 0.001
3	4	50	**–**	**–**		7.41	(1.563~35.110)	0.012
TNM_M stage					< 0.001			
No	161	97.5	88.64	85.44		1		
Yes	14	53.85	30.77	**–**		5.24	(1.876~14.649)	0.002

## Discussion

In this study, we found significantly better DFS and OS for dMMR subtype patients compared with pMMR subtype patients, although both were classified clinically as HNPCC. Furthermore, similar to LS and FCCTX have different phenotypic features, we found significant differences existing between dMMR and pMMR subtype patients, as summarized in Table 5, a review of the literature [6, 13–16], including age at time of CRC diagnosis, tumor location, rate of metachronous CRC, and rate of extra-colonic secondary tumors. As shown in Table 1, the age at time of CRC diagnosis was 6.3 years older for pMMR. There was a right colon predominance (61.7% vs. 27.3%) for dMMR patients compared to a rectal tumor predominance (41.8% vs. 11.7%) for pMMR patients. Furthermore, significantly higher frequencies of secondary-site primary tumors (16.7% vs. 1.8%) and metachronous CRC (20.57 person-year vs. 6.18 person-year) were found in dMMR patients compared to pMMR patients. Moreover, we found significantly more N0 tumors in dMMR (70.8%) compared with pMMR (50.9%) (*p* = 0.049), as shown in Table 1.

**Table 5:** Summary of different phenotypes between Lynch syndrome (LS) and familial colorectal cancer type X (FCCTX) in the literature

	Age at diagnosis of CRC	Tumor location	Rate of meta-chronous CRC	Rate of extra-colonic 2^**nd**^ tumor
	LS vs. FCCTX	LS vs. FCCTX	LS vs. FCCTX	LS vs. FCCTX
**Valle** [13]	Mean 41 vs. 53 years	Right colon	26.3% vs. 7.7%	No data
	*p* < 0.001	70.6% vs. 20.8%	NS	
		Rectum		
		11.8% vs. 41.7%		
**Mueller-Koch** [14]	Median 41 vs. 55 years	Right colon	11.3% vs. 1.4%	16% vs. 1.4%
	*p* < 0.001	68% vs. 14%	*p* = 0.017	*p* < 0.001
		*p* < 0.010		
**Xavier L** [15]	64.8 vs. 67.8 years	Right colon	No data	5.1% vs. 3.3%
	*p* = 0.603	44.4% vs. 13.3%		NS
		*p* = 0.15		
**Shiovitz** [6]	Mean 53.3 vs. 50.5 years	Right colon	No data	No data
		61% vs. 31%		
		Rectum		
		15% vs. 34%		
**Yamaguchi** [16]	Median 48 vs. 45 years	Right colon	No data	37.6% vs. 4%
	*p* = 0.39	56% vs. 62%		*p* = 0.001
**This study**	Mean 54.3 vs. 48.6 years	Right colon	8.3% vs. 1.8%	16.7% vs. 1.8%
	*p* = 0.007	62% vs. 27%	*p* = 0.177	*p* = 0.005
		Rectum		
		12% vs. 42%		
		*p* < 0.001		

*NS* not significant

In addition to noting different phenotypes between dMMR and pMMR subtype patients, this retrospective study determined that extended colectomy was performed more often for dMMR than pMMR patients (35.8% vs. 14.6%, *p* = 0.004). Most studies recommend extended resection, such as subtotal or total colectomy rather than segmental resection or hemicolectomy, for the surgical treatment of dMMR or LS patients because of the high risk of metachronous CRC [14, 17–19]. However, no prospective or randomized study has demonstrated that extended resection confers a survival benefit compared with segmental resection for HNPCC patients. Furthermore, little is known about the ideal operation type may be tailored according to MMR status. In this study, we determined that the risk of metachronous colorectal cancer in pMMR subtype was significantly lower than that of dMMR subtype and comparable to that of sporadic CRC. Furthermore, in pMMR subtype patients, the tumor was more frequently located in the rectum, and extended colectomy, which was only performed in 14.6% of these patients, was not the preferred surgical treatment. However, segmental colectomy itself for pMMR patients did not significantly affect surgical outcomes and was not an independent factor for OS and DFS. Clinically, these two disease subtypes are both classified as HNPCC; the inherent difficulty lies in distinguishing the two because preoperative genetic testing such as microsatellite instability or dMMR status are not always available in daily clinical practice and may affect surgical decision making. However, surgeons have some clues that suggest pMMR subtype patients, such as older age (average age 54.3 years in this study), less right colon involvement (27.3% in this study), and a higher rate of rectal involved (41.8% in this study) [13, 15]. Thus, extended colectomy might be recommended for HNPCC patients without rectal tumor involvement and age younger than 50 years because of the higher rate of dMMR subtype with these features. In contrast, segmental resection might be recommended for HNPCC patients who present with rectal cancer and older age due to the high rate of pMMR with these features.

However, for all HNPCC patients, post-operation MMR status should be routinely checked. Post-operative colonoscopy and extra-colonic surveillance were thus individualized based on MMR data [17–19]. Post-operation frequent colonoscopy surveillance of dMMR subtype patients who undergo segmental or hemicolectomy is recommended because the risk of metachronous CRC becomes an important issue, as shown in this study (6.18 for pMMR patients and 20.57 person-years for dMMR patients, *p* < 0.001). In this study, pMMR patients had a low rate of metachronous colorectal cancer, and 85% of these cases underwent segmental colectomy; this highlights the idea that as respect to pMMR patients might benefit from a follow-up program similar to that of sporadic CRC, because of the low incidence of secondary-site tumors. In addition, because of the low rate of secondary-site cancer in pMMR, the post-operative surveillance of these patients might target the CRC only, with a longer interval (such as 3–5 years) [16, 18]. However, for dMMR patients, postoperative surveillance of secondary-site cancers (15.0% vs. 5.3%) should be emphasized. Clinically, routine determination of post-operative tumor MMR status is strongly recommended to tailor surveillance programs and improve outcomes.

## Limitations

This analysis benefits from the use of a cohort of patients with standardized computerized data collection, providing the opportunity to compare dMMR and pMMR subtypes for detailed analysis of clinicopathologic variables. However, the major limitation of this study is its retrospective nature. This study did not involve universal screening of mismatch repair proteins using immunohistochemical staining. Instead, screening was performed for cases whose family history fulfilled the A-II criteria. Therefore, few cases were missed in this cohort.

## Conclusion

In addition to several distinct clinicopathological features existing between dMMR and pMMR subtypes patient, we found more N0 cases, more cases of extended colectomy, and significantly better DFS and OS for dMMR patients compared with pMMR patients. Postoperative tumor MMR status that is highly recommended routinely performed to tailor surveillance programs because of the significantly different risk of metachronous CRC and secondary-site tumors.

## Data Availability

The detailed patients’ databases generated and analyzed during this study are not publicly available due to appropriate protection of patients’ personal information but are available from the corresponding author on reasonable request.

## Abbreviations

CRC: Colorectal cancer
HNPCC: Hereditary non-polyposis colorectal cancer
pMMR: Proficient mismatch repair gene expression
dMMR: Deficient mismatch repair gene expression
DFS: Disease-free survival
OS: Overall survival
LS: Lynch syndrome
FCCTX: Familial colorectal cancer type X
